# Editorial on Special Issue “Quantitative PET and SPECT”

**DOI:** 10.3390/diagnostics12081989

**Published:** 2022-08-16

**Authors:** Floris H. P. van Velden, Lioe-Fee de Geus-Oei

**Affiliations:** 1 Section of Nuclear Medicine, Department of Radiology, Leiden University Medical Center, 2333 ZA Leiden, The Netherlands; 2 Biomedical Photonic Imaging Group, University of Twente, 7522 NB Enschede, The Netherlands

Since the introduction of personalized (or precision) medicine, where individually tailored treatments are designed to deliver the right treatment to the right patient at the right time, the primary focus of imaging has moved from detection and diagnosis to tissue characterization, determination of prognosis, prediction of treatment efficacy, and measurement of treatment response [1]. Precision (personalized) imaging relies heavily on the use of hybrid imaging technologies and a variety of quantitative imaging biomarkers. The growing number of promising theragnostics, treatment strategies that combine radiolabeled therapeutics with diagnostics, require accurate quantification for pre- and post-treatment dosimetry. Furthermore, quantification is essential in the pharmacokinetic analysis of promising new radiotracers and drugs, and in the assessment of drug resistance. This Special Issue highlights trending research topics of two quantitative imaging tools used in nuclear medicine, positron emission tomography (PET) and single photon emission computed tomography (SPECT).

PET is by nature a quantitative imaging tool, relating the time–activity concentration in tissues and the basic functional parameters governing the biological processes being studied. The quantitative accuracy and interpretation of PET images are influenced by many factors, which are summarized in the review of Rogasch and colleagues, emphasizing the need to implement quality control and standardized imaging protocols [2]. In this Special Issue, three research articles address factors influencing quantitative accuracy and/or interpretation of PET images, such as image reconstruction. Based on the evaluation of multiple Bayesian penalized likelihood (BPL) reconstructions of phantom scans, Rijnsdorp et al. identified the optimal noise penalty factor for BPL reconstruction of clinical ^68^Ga-PSMA PET/CT scans in terms of detectability and reproducibility [3]. A second factor impacting the quantitative accuracy of PET is the spatial resolution, which is influenced by, amongst others, the positron range of the imaged isotope. In this Special Issue, Yang developed a convolutional neural network, originally designed to convert magnetic resonance imaging (MRI) into pseudo computed tomography (CT) scans, to correct for the positron range in preclinical ^68^Ga-PET imaging [4]. Third, respiratory motion degrades the quantification accuracy of PET imaging [5] and, when left uncorrected, could thereby impact the evaluation of selective internal radiation therapy (SIRT) dosimetry. Walker and colleagues showed that post-therapy ^90^Y SIRT PET/CT imaging, in terms of tumor quantification and dosimetric measures, is improved by quiescent period respiratory motion correction [6].

Recent innovations in SPECT reconstruction techniques have allowed SPECT to move from relative/semi-quantitative measures to absolute quantification [7]. So far, absolute SPECT is only limitedly translated to diagnostic nuclear medicine, requiring proper validations with a ground truth, such as imaging phantoms. The review by De Schepper and colleagues showed that these validations are currently feasible with the use of application-specific phantoms produced by the current state-of-the-art in additive manufacturing or 3D printing [8].

The strength of PET and SPECT is that they permit whole-body molecular imaging in a noninvasive way, evaluating multiple disease sites. In this Special Issue, Iqbal et al. investigated the diagnostic accuracy of ^18^F-FDG PET for staging of patients with grade 1–2 estrogen receptor positive (ER+) breast cancer and showed that ^18^F-FDG PET inadequately staged almost 30% of these patients, illustrating the urgent need for new radiotracers to improve the current imaging staging procedures for these patients [9]. 

Serial scanning allows the measurement of functional changes over time during therapeutic interventions. In this Special Issue, Roef and colleagues determined the repeatability of ^68^Ga-PSMA lesion uptake in both relapsing and metastatic tumors and showed that a minimum response of 50% seems appropriate in this clinical situation [10], which is higher than 30% recommended by the PET Response Criteria in Solid Tumors [11]. In this Special Issue, three articles investigated the prognostic value of ^18^F-FDG PET imaging. Chen et al. investigated whether the combination of primary tumor and nodal PET parameters can predict survival outcomes in patients with nodal metastatic non-small cell lung cancer without distant metastasis and demonstrated that by using this combination of PET features (in specific a combination of total lesion glycolysis values; TLG) with clinical factors risk stratification can be refined, facilitating tailored therapeutic strategies for these patients [12]. In addition, Kalisvaart and colleagues determined the added prognostic value of PET features in patients with metastases from soft tissue sarcoma, identifying the maximum and peak standardized uptake values as independent prognostic factors for overall survival in these patients [13]. Finally, Hlongwa et al. demonstrated that high whole-body TLG, and metabolic tumor volumes and TLG of the primary tumor were prognostic factors for overall survival in patients with malignant melanoma [14].

Images can no longer be treated strictly as pictures but instead must use innovative approaches based on numerical analysis. Medical images contain much more information hidden in the millions of voxels that cannot be assessed by the human eye. Recent developments in computer science have introduced computational methods that can capture this concealed information, which is studied in the field of radiomics that includes (a variety of) quantitative imaging biomarkers. Radiomics have the potential to improve knowledge of tumor biology and, combined with clinical data and other biomarkers, guide clinical management decisions, thereby contributing to precision medicine [15]. Currently, there is no consensus regarding the inclusion of regions of central necrosis during tumor delineation for radiomic analysis. In this Special Issue, Noortman and colleagues showed that central necrosis of tumors on ^18^F-FDG PET significantly impacts radiomic feature values but did not seem to impact the predictive performance of the radiomics model [16].

Only with a dynamic scan is it possible to follow the kinetics (uptake, retention, clearance) of the radiotracer quantitatively [17]. However, the pharmacokinetic analysis often requires an arterial input function (AIF) that is acquired by an invasive arterial blood sampling procedure. As a noninvasive surrogate to the AIF, Fang et al. developed an image-derived input function using a model-based matrix factorization to measure the volume of distribution that quantifies the 18-kDa translocator protein (TSPO) of ^18^F-DPA-714 PET in the human brain [18]. In another, more exploratory and noninvasive dynamic PET study to assess the presynaptic dopamine synthesis capacity using ^18^F-FDOPA in the human brain, Schalbroeck et al. revealed that, among autistic adults, specific autistic traits can be associated with reduced striatal dopamine synthesis capacity [19]. Last but not least, this Special Issue also identifies a potential role for dynamic PET to monitor treatment response in smoldering myeloma using ^18^F-FDG, as illustrated by a case report by Sachpekidis et al. [20].

In conclusion, the manuscripts published in this Special Issue highlight hot topics on quantitative PET and SPECT, discussing developments in the field of radiomics, the rise of artificial intelligence techniques, and the problems that have to be solved to be able to move towards validated and clinically accepted quantitative imaging biomarkers for precision medicine. We would like to sincerely thank all authors for their contributions and hope that the readers will enjoy reading this Special Issue.
